# Embolization of the Middle Meningeal Artery As Primary Treatment for Chronic Subdural Hematoma in Older Filipino Adults

**DOI:** 10.7759/cureus.97406

**Published:** 2025-11-21

**Authors:** Jan Erik B Detran, Jay B Villavicencio

**Affiliations:** 1 Department of Surgery, Rizal Medical Center, Pasig City, PHL; 2 Section of Neurosurgery, Department of Neurosciences, Makati Medical Center, Makati City, PHL

**Keywords:** chronic subdural hematoma, filipino, low- and middle-income country, middle meningeal artery embolization, neurosurgery, philippines

## Abstract

Chronic subdural hematoma (CSDH) is among the most common neurosurgical conditions in older adults, traditionally managed with burr-hole drainage, which may be associated with recurrence and perioperative risks. Embolization of the middle meningeal artery (EMMA) has recently emerged as a minimally invasive alternative with promising safety and efficacy profiles.

This case series describes the institutional experience with EMMA as a primary treatment for CSDH in older Filipino adults managed in a government tertiary hospital. The majority of patients demonstrated radiographic improvement, and most showed clinical recovery without major complications. Anatomical factors, such as vessel caliber and completeness of branch embolization, appeared to influence treatment outcomes.

These early findings suggest that EMMA is a feasible and potentially effective option for older adults with CSDH, particularly for those with comorbidities that increase surgical risk. Larger prospective studies are warranted to further define its role in low- and middle-income settings.

## Introduction

Chronic subdural hematoma (CSDH) is one of the most common neurosurgical conditions worldwide, characterized by a liquefied hematoma confined within the potential subdural space [1]. It typically arises from minor inertial brain injury that causes tearing of bridging veins in the dural border cell layer, leading to slow bleeding and hematoma formation [1]. Ongoing microhemorrhage from fragile, inflamed neomembranes - supplied primarily by the middle meningeal artery (MMA) - sustains the hematoma [1]. These membranes consist of thin-walled capillaries with abundant endothelial gaps that permit plasma extravasation, perpetuating inflammation, fibrinolysis, and recurrent bleeding in a self-reinforcing cycle of hematoma expansion [1,2]. An additional mechanism involves osmotic and oncotic pressure gradients between the hematoma and cerebrospinal fluid, driving further exudation and transudation through the neomembranes [1]. On non-contrast computed tomography (NCCT), CSDH evolves from hypodense or hyperdense lesions in the acute phase to mixed and ultimately hypodense lesions as it matures and resolves [1]. Clinically, patients often present with progressive cognitive decline, headache, or focal neurological deficits such as paresis or seizures [1,2]. Established risk factors include advanced age, anticoagulant or antiplatelet use, brain atrophy, chronic alcohol use, neurodegenerative and systemic diseases, and procedures causing dural injury such as shunt insertion, lumbar puncture, or spinal surgery [1].

In general, small, asymptomatic CSDHs are managed conservatively [2]. In contrast, patients with symptomatic or large hematomas are typically treated surgically through twist drill craniostomy, burr hole craniostomy, or craniotomy [1,2]. Occasionally, a negative pressure system, such as a subdural evacuating port, is used as an adjunct [1]. These interventions carry a risk of periprocedural complications and possible CSDH recurrence [2]. Recurrence is defined as the appearance of new or recurrent preoperative symptoms within six months after surgery [1]. According to a 2012 meta-analysis, the recurrence rates of craniotomy, twist drill craniostomy, and burr hole craniostomy were 3.9%, 2.5%, and 9.3%, respectively, while the highest mortality rate of 12.2% was associated with craniotomy [2].

More recently, embolization of the middle meningeal artery (EMMA) has emerged as a minimally invasive alternative to surgical evacuation [3], demonstrating significantly lower recurrence rates ranging from 1.4% to 8.9% [4]. This growing evidence is supported by major clinical trials such as EMBOLISE (Embolization of the Middle Meningeal Artery With Onyx Liquid Embolic System in the Treatment of Subacute and Chronic Subdural Hematoma), MAGIC-MT (Middle Meningeal Artery Treatment), and STEM (Squid Trial for the Embolization of the MMA for the Treatment of CSDH) [5]. By targeting and occluding the abnormal neovasculature that sustains the hematoma, middle meningeal artery embolization promotes resorption while preserving normal venous outflow, with the goal of achieving durable vascular occlusion to minimize recurrence [6].

The multicenter EMBOLISE trial enrolled 400 predominantly older adults (mean age in the mid-70s) with symptomatic subacute or chronic SDH requiring surgical evacuation and found that adjunctive EMMA significantly reduced recurrence or progression, leading to repeat surgery compared with surgery alone [7]. In the large MAGIC-MT trial conducted across 31 centers in China, 722 patients (mean age 69 years) with nonacute CSDH - most of whom underwent burr-hole drainage - showed similar recurrence rates between groups, though EMMA was associated with fewer serious adverse events [8]. Likewise, the STEM trial, which included 310 patients (mean age 73 years, 70% male) receiving either surgical or nonsurgical standard treatment, demonstrated that adjunctive EMMA significantly lowered treatment failure rates without increasing disabling stroke or mortality [6]. Collectively, these studies provide strong evidence supporting the efficacy and safety of EMMA across diverse older adult populations. Although global data on EMMA outcomes are becoming increasingly robust, knowledge gaps and practical nuances in low- and middle-income countries remain largely unexplored, particularly among elderly patients. This case series aims to describe our institutional experience with EMMA as a primary treatment for CSDH in older Filipino adults.

Older adults with CSDH often present with multiple comorbidities, including cardiovascular, pulmonary, and metabolic conditions, that increase the risks associated with general anesthesia and open surgical procedures. For these patients, burr hole drainage under general anesthesia may pose greater harm than benefit. In contrast, EMMA, as a less invasive intervention, allows neurosurgeons to mitigate these perioperative risks while still achieving hematoma resolution.

In the Philippines, CSDH accounts for the largest burden (35.2%) of neurosurgical diseases among geriatric patients (aged ≥65 years), with burr hole drainage being the most commonly performed neurosurgical procedure in this population (34.8%) [9]. To the authors’ knowledge, this is the first Philippine case series to report on older Filipino adults with CSDH treated primarily with EMMA, following the Barrow algorithm [2], in a tertiary government hospital setting.

This study aims to (1) identify patient risk factors for CSDH, (2) describe the endovascular technique utilized, and (3) assess radiographic and clinical outcomes. Radiographic success is defined as a post-EMMA reduction in CSDH thickness of at least 50% compared to the pre-EMMA maximum thickness on cranial NCCT axial views. Reduction below 50%, unchanged thickness, or interval increase is classified as radiographic failure. Clinical success is defined as a decrease in the modified Rankin Scale (mRS) score post-EMMA compared to baseline. Unchanged functional outcome refers to an identical mRS score before and after EMMA, while clinical deterioration is indicated by an increased mRS post-procedure. This case series is reported in accordance with the Preferred Reporting Of CasE Series in Surgery (PROCESS) 2023 guidelines [10].

## Case presentation

Patient selection and statistical analysis

**Figure 1:**
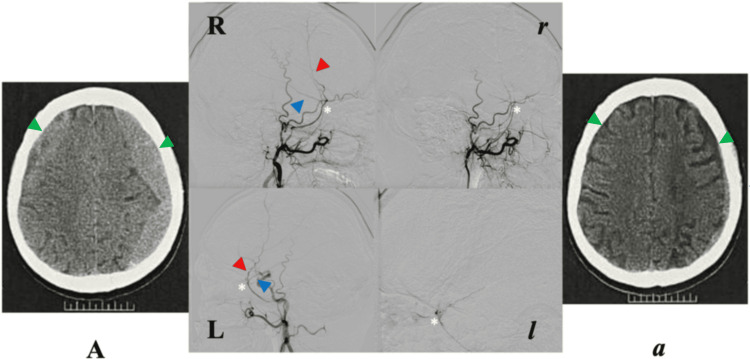
Case A. Pre-EMMA (A) and respective post-EMMA (a) cranial NCCT axial views at levels of maximum CSDH thickness. Pre-EMMA angiographies in lateral projections (R, right; L, left) show the course of anterior and posterior MMA branches, and post-EMMA obliteration of these vessels (r, right; l, left). Angiographic projections correspond to contiguous NCCTs. EMMA, embolization of the middle meningeal artery; L, left; MMA, middle meningeal artery; NCCT, non-contrast computed tomography; R, right. Green arrowhead, chronic subdural hematoma; red arrowhead, anterior branch; blue arrowhead, posterior branch; white asterisk, MMA bifurcation point.

**Figure 2:**
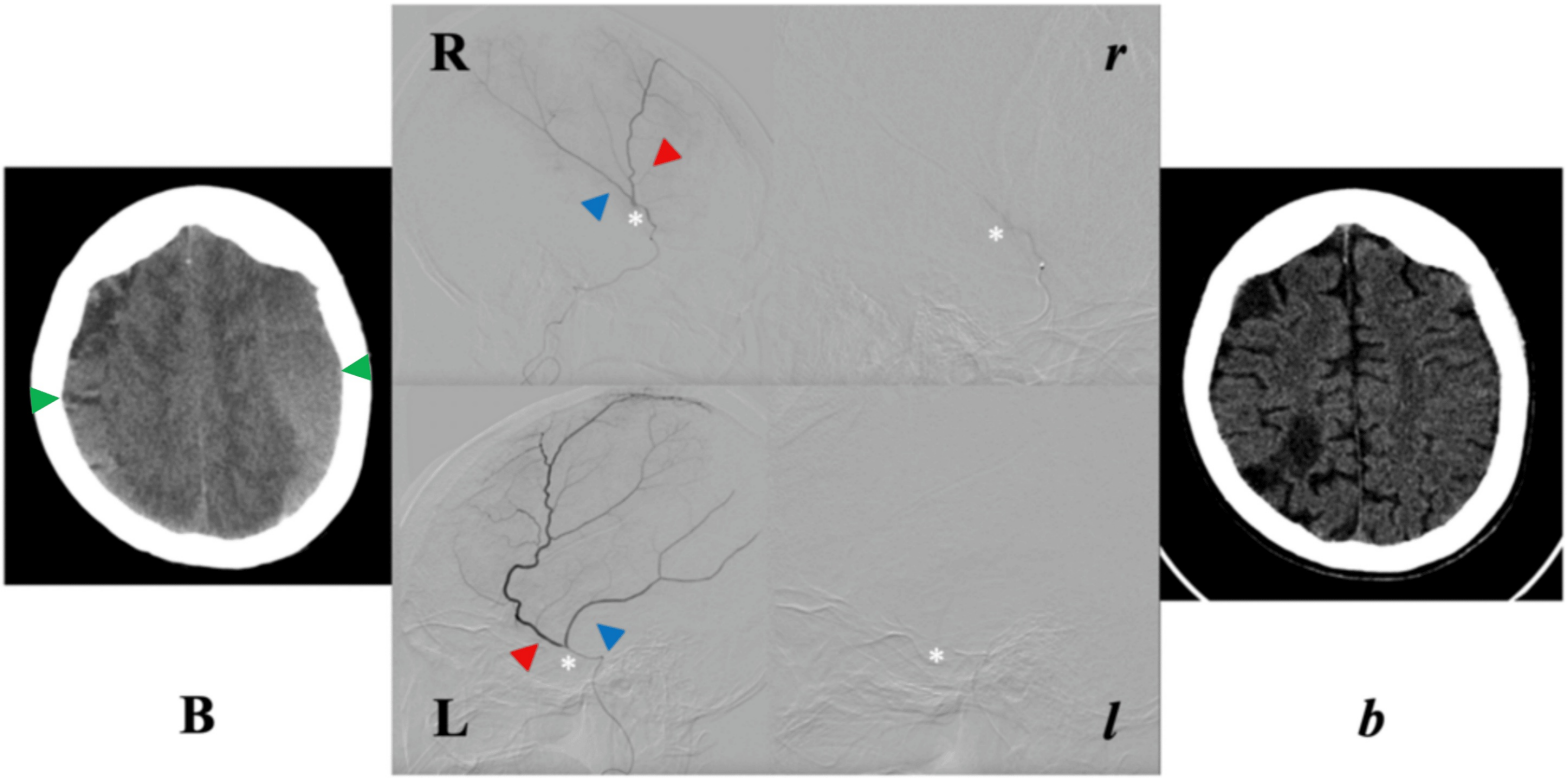
Case B. Pre-EMMA (B) and respective post-EMMA (b) cranial NCCT axial views at levels of maximum CSDH thickness. Pre-EMMA angiographies in lateral projections (R, right; L, left) show the course of anterior and posterior MMA branches, and post-EMMA obliteration of these vessels (r, right; l, left). Angiographic projections correspond to contiguous NCCTs. EMMA, embolization of the middle meningeal artery; L, left; MMA, middle meningeal artery; NCCT, non-contrast computed tomography; R, right. Green arrowhead, chronic subdural hematoma; red arrowhead, anterior branch; blue arrowhead, posterior branch; white asterisk, MMA bifurcation point.

**Figure 3:**
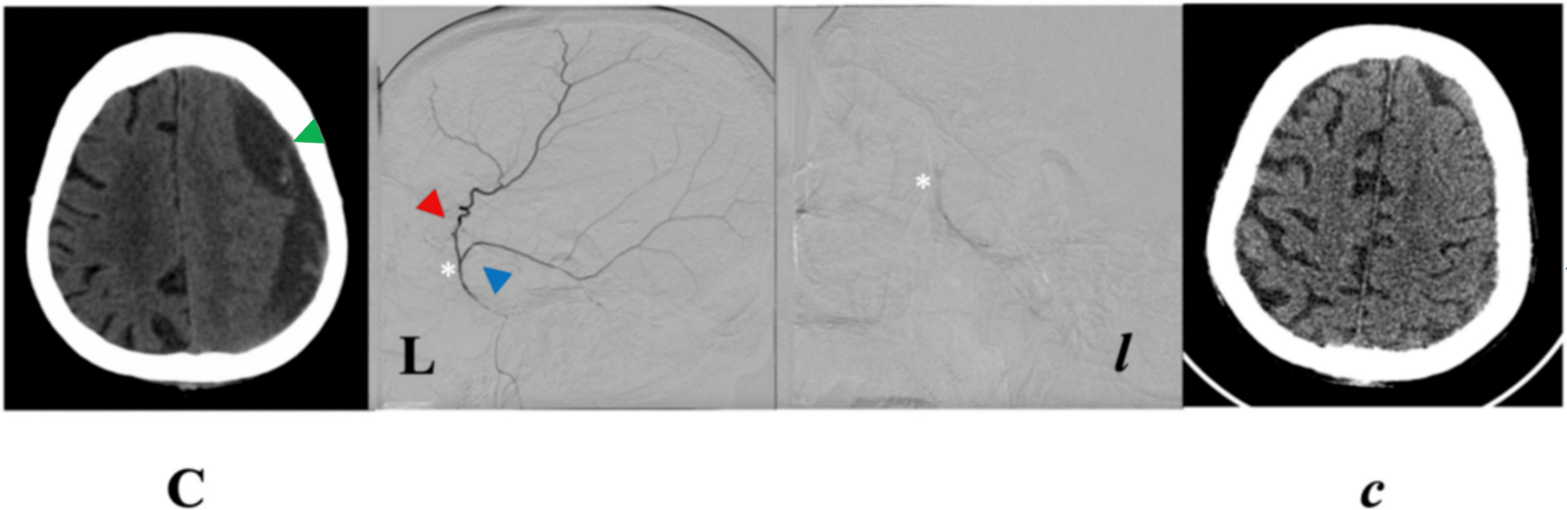
Case C. Pre-EMMA (C) and respective post-EMMA (C) cranial NCCT axial views at levels of maximum CSDH thickness. Pre-EMMA angiography in left lateral projection (L) shows the course of anterior and posterior MMA branches, and post-EMMA obliteration of these vessels (l). Angiographic projections correspond to contiguous NCCTs. EMMA, embolization of the middle meningeal artery; MMA, middle meningeal artery; NCCT, non-contrast computed tomography; R, right. Green arrowhead, chronic subdural hematoma; red arrowhead, anterior branch; blue arrowhead, posterior branch; white asterisk, MMA bifurcation point.

**Figure 4:**
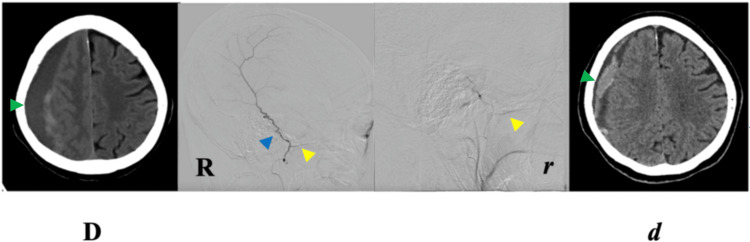
Case D. Pre-EMMA (D) and respective post-EMMA (d) cranial NCCT axial views at levels of maximum CSDH thickness. Pre-EMMA angiography in right lateral projection (R) shows the course of posterior MMA branch and absence of anterior MMA branch, and post-EMMA obliteration of the posterior branch (r). Angiographic projections correspond to contiguous NCCTs. EMMA, embolization of the middle meningeal artery; MMA, middle meningeal artery; NCCT, non-contrast computed tomography; R, right. Green arrowhead, chronic subdural hematoma; blue arrowhead, posterior branch; yellow arrowhead, sphenoidal branch.

Four consecutive patients with CSDH who underwent endovascular EMMA at Rizal Medical Center between June and August 2024 were included in this case series. Written informed consent was obtained from all patients or their legal guardians. Demographic, clinical, and radiologic data were retrospectively collected from institutional records. None of the patients had undergone prior surgical evacuation for CSDH. All underwent post-procedural clinical and radiographic follow-up (Table 1; Figures 1-4).

**Table 1:** Demographics, pre-EMMA status, risk stratification, intraprocedural data, and post-EMMA status of patients in case series ACS, acute coronary syndrome; AF, atrial fibrillation; ARISCAT, Assess Respiratory Risk in Surgical Patients in Catalonia; BP, branch point; BPPV, benign paroxysmal positional vertigo; CVD, cerebrovascular disease; EMMA, embolization of the middle meningeal artery; HTN, hypertension; mRS, modified Rankin Scale; PTB, pulmonary tuberculosis; PVA, polyvinyl alcohol; RCRI, Revised Cardiac Risk Index; T2DM, type 2 diabetes mellitus.

Patient (Age/Sex)	A (63/M)	B (70/F)	C (86/F)	D (71/F)
Demographics and Pre-EMMA Status				
Comorbidities	Chronic cerebrovascular disease (CVD) infarct, hypertension (HTN), type 2 diabetes mellitus (T2DM), dyslipidemia	Acute coronary syndrome (ACS), chronic CVD infarct, HTN, T2DM	Atrial fibrillation (AF) in cardiovascular risk (CVR), HTN, dyslipidemia, pulmonary tuberculosis (PTB)	Benign paroxysmal positional vertigo (BPPV), PTB
Antiplatelet Use	Clopidogrel	Aspirin, clopidogrel	None	None
Anticoagulant Use	None	None	Apixaban	None
Statin Use	Rosuvastatin	Atorvastatin	Rosuvastatin	None
Alcohol Use	Yes	None	None	None
Recent Head Trauma (Weeks Post-Injury)	Motor vehicle accident (7)	None (n/a)	None (n/a)	Mauling (4)
Pre-EMMA Signs/Symptoms	Cognitive decline, right hemiparesis	Right hemiparesis, headache	Decreased sensorium, cognitive decline	Headache
mRS Grade on Admission	2	3	5	1
Risk Stratification, Intraprocedural Data, and Post-EMMA Status				
Risk for Cardiopulmonary Complications	Intermediate	Intermediate	Intermediate	Intermediate
RCRI Class	II	II	II	II
ARISCAT Score	26	37	16	26
IV Sedation	Midazolam, fentanyl	Midazolam, fentanyl	Midazolam, fentanyl	Fentanyl
Access	Right transfemoral	Right transfemoral	Right transfemoral	Right transradial
Embolic Agent	PVA	PVA	PVA	PVA
Laterality and Duration (min)	Bilateral, 165	Bilateral, 67	Left, 45	Right, 80
Puncture Site Hematoma	No	Yes	Yes	No
Total Hospital Days (vs. Post-EMMA Days Prior to Discharge)	7 (2)	28 (8)	15 (4)	5 (3)
Post-EMMA Signs/Symptoms	Persistent cognitive decline, improved hemiparesis	Improved hemiparesis, improved headache	Improved sensorium, improved cognition	Persistent headache
mRS Grade on Latest Follow-Up (Weeks Post-EMMA)	2 (8) - No change	1 (12) - Improved	2 (8) - Improved	1 (6) - No change

Statistical analysis was performed using DATAtab (DATAtab e.U., Graz, Austria). Hematoma thickness values before and after embolization were compared primarily using the Wilcoxon signed-rank test, appropriate for small, non-normally distributed paired samples (n = 6). For completeness, a paired t-test and a t-based 95% confidence interval (CI) for the mean difference were also calculated. Descriptive statistics were expressed as mean ± standard deviation (SD) and median with range. Statistical significance was defined as p < 0.05. Given the small cohort, all findings are exploratory and intended to highlight trends rather than to provide definitive inferential conclusions.

EMMA protocol

The EMMA of all four patients was performed by the same consultant endovascular neurosurgeon, assisted by one or two neurosurgery residents. The Shimadzu Trinias Angiography System was the equipment utilized in the hospital catheterization laboratory. The procedure begins with the patient in a supine position under intravenous sedation. Arterial puncture at the right radial (radial artery) or femoral (common femoral artery) site is done using G20 or G22, or G18 needle, respectively, via the Seldinger technique. A 6-French transradial or femoral introducer sheath was inserted and connected to a pressurized, heparinized saline drip. A 5-French Simmons catheter was inserted and placed into the left or right internal carotid artery. Using an exchange length wire, the Simmons catheter was replaced with a 7-French guider Softip XF. Through the guider, a renegade microcatheter was inserted and placed into the MMA just proximal to its bifurcation into the anterior and posterior branches. Contour polyvinyl alcohol (PVA) particles (150-250 µm), admixed with contrast solvent (iohexol or iopamidol), were slowly instilled in a pulsatile manner through the microcatheter until the serial post-EMMA angiogram showed obliteration of the frontal and parietal branches (Figures 1-4). For bilateral lesions, the Simmons catheter is repositioned into the contralateral internal carotid artery, with the same aforementioned steps for contralateral EMMA embolization and serial post-EMMA angiogram. The microcatheter was then pulled out, followed by the sheath. Puncture site was manually compressed for 15 to 30 minutes, and was covered with a compact gauze dressing with a transradial band for 12 hours (radial) or sandbag weights for six hours (femoral). These steps were done in accordance with the consultant endovascular neurosurgeon’s discretion and institutional protocols.

Results

Patient Demographics and Procedural Data

Patients ranged in age from 63 to 86 years (mean 72.5 ± 9.68), with one male and three females. Pre-embolization mRS scores ranged from 1 to 5 (mean 2.75 ± 1.71). Pre-EMMA hematoma thickness ranged from 20 to 30 mm (mean 22.17 ± 3.92 mm). MMA trunk diameters measured 1.27-1.92 mm (mean 1.59 ± 0.24 mm), and no dangerous collaterals were observed on proximal external carotid angiograms (Tables 2-3; Figures 1-4).

**Table 2:** Radiographic features of CSDH before and after EMMA CSDH, chronic subdural hematoma; EMMA, embolization of the middle meningeal artery; MLS, midline shift. Arrows (→) indicate the direction of MLS; R, right; L, left. Percent reduction = (Pre - Post) / Pre × 100.

Age/Sex	CSDH Laterality	Pre-EMMA Weeks Post-Injury	Location	Pre-EMMA Nakaguchi/Nomura Classification	Pre-EMMA Max Thickness (mm)	Pre-EMMA MLS (mm)	Weeks Post-EMMA	Post-EMMA Location	Post-EMMA Nakaguchi/Nomura Classification	Post-EMMA Max Thickness (mm) (% Reduction)	Post-EMMA MLS (mm)
63/M	Left	7	Frontal/Parietal	Hyperdense homogeneous	21	0	8	Frontal/parietal	Hyperdense homogeneous	7 (66.7%)	0
63/M	Right	7	Frontal/parietal	Mixed densities laminar	20	0	8	Frontal/parietal	Hypodense homogeneous	3 (85.0%)	0
70/F	Left	n/a	Frontal/parietal	Mixed densities trabecular	20	5 → R	12	None	n/a	0 (100%)	0
70/F	Right	n/a	Frontal/parietal	Mixed densities trabecular	20	5 → R	12	None	n/a	0 (100%)	0
86/F	Left	n/a	Frontal/parietal	Mixed densities laminar	22	9 → R	8	None	n/a	0 (100%)	0
71/F	Right	4	Frontal/parietal	Mixed densities laminar	30	5 → L	6	Frontal/parietal	Mixed densities laminar	23 (23.3%)	3 → L

**Table 3:** Angiographic middle meningeal artery (MMA) morphometry and procedural parameters BP, branch point of MMA; EMMA, embolization of the middle meningeal artery; IMA, internal maxillary artery; MMA, middle meningeal artery; n/a, not applicable due to absent anterior branch and posterior-only embolization. “Origin-BP length” denotes the distance from the origin of the middle meningeal artery (MMA) at the internal maxillary artery (IMA) to the branch point of the MMA, where it bifurcates into anterior and posterior branches. “Catheter distal point” denotes the distance from the MMA branch point, where the embolic agent was deployed from the catheter. Branch dominance on angiographic lateral projection is defined as follows: • Anterior – most secondary branches are located anterior to the anterior branch. • Codominant – most secondary branches are found between the anterior and posterior branches. • Posterior – most secondary branches are located posterior to the posterior branch. EMMA Grade 3 indicates that both anterior and posterior branches of the MMA were completely embolized, with no obvious meningeal parenchymal blush seen on the proximal external carotid artery (ECA) trunk angiogram at the end of embolization.

Patient	MMA Laterality	Origin	Trunk Thickness (mm)	Origin-BP Length (mm)	Anterior Branch Thickness (mm)	Posterior Branch Thickness (mm)	Dominant Branch	Catheter Distal Point (mm)	EMMA Grade
A	Left	IMA	1.45	33.51	1.2	1.2	Anterior	9.73 proximal to BP (nonselective)	3
A	Right	IMA	1.71	55.84	1.34	1.08	Anterior	3.44 proximal to BP (nonselective)	3
B	Left	IMA	1.92	42.99	1.06	1.22	Codominant	3.77 proximal to BP (nonselective)	3
B	Right	IMA	1.42	12.91	0.93	0.86	Codominant	10.10 proximal to BP (nonselective)	3
C	Left	IMA	1.8	57.85	1.22	0.99	Codominant	7.89 proximal to BP (nonselective)	3
D	Right	IMA	1.27 (proximal to sphenoidal branch)	n/a	n/a	1.27 (distal to sphenoidal branch)	Posterior-only	Distal to sphenoidal branch, no BP (superselective)	n/a

Post-embolization, hematoma thickness decreased across all patients, ranging from 0 to 23 mm (mean 5.50 ± 9.01 mm), corresponding to a mean reduction of 16.67 ± 5.50 mm (median 18.5 mm; range 7-22 mm). The Wilcoxon signed-rank test demonstrated a significant reduction (p = 0.0313), and the paired t-test yielded t(5) = 7.42, p = 0.0007. The 95% CI for the mean difference (10.89-22.44 mm) supports a consistent reduction trend. These results should be interpreted cautiously, given the small sample.

Radiographic resolution paralleled neurological improvement in three of four patients (75%), while one patient (Case D) showed only partial radiographic and clinical response due to limited posterior-only embolization (Table 4; Figure 4). Minor puncture-site hematomas were observed in two patients and resolved spontaneously. No intracranial or systemic embolic complications occurred, and no patients experienced new or worsening neurological deficits. Post-EMMA mRS scores ranged from 1 to 2 (mean 1.5 ± 0.58). Individual data are summarized in Tables 1-3 and Figures 1-4.

**Table 4:** Subgroup patterns of demographics, pre-EMMA status, risk stratification, and post-EMMA follow-up intervals in chronic subdural hematoma patients (four patients, six hematoma sides) CSDH, chronic subdural hematoma; EMMA, embolization of the middle meningeal artery; RCRI, Revised Cardiac Risk Index; ARISCAT, Assess Respiratory Risk in Surgical Patients in Catalonia; Radiographic Success = ≥50% reduction in hematoma thickness on follow-up non-contrast computed tomography (n = six hematoma sides); Clinical Improvement = ≥1-grade reduction in modified Rankin Scale or resolution of presenting neurological symptoms (n = four patients). Cardiovascular comorbidities include cerebrovascular disease, hypertension, and ischemic heart disease.

Subgroup	Number of Patients	Radiographic Success (≥ 50%)	Clinical Improvement	Notes/Observations
Age - < 75 years	3	4/4	1/3	Patients A and D unchanged; B improved clinically; D radiographic failure on one side
Age - ≥ 75 years	1	2/2	1/1	Patient C improved clinically; full embolization
Sex - Male	1	2/2	0/1	Patient A unchanged clinically despite radiographic success
Sex - Female	3	3/4	2/3	B, C improved; D unchanged; D radiographic failure
Cardiovascular Comorbidity	3	4/5	2/3	A, B, C had comorbidities; D unchanged
No Cardiovascular Comorbidity	1	0/1	0/1	D partial response, posterior-only embolization
Antiplatelet Use	2	3/3	1/2	A, B on dual therapy; A unchanged clinically
No Antiplatelet	2	1/3	1/2	C improved; D unchanged
Anticoagulant Use	1	2/2	1/1	C on apixaban; full radiographic and clinical improvement
No Anticoagulant	3	2/4	2/3	Partial response in D
Statin Use	3	4/5	2/3	A, B, C; D non-user, unchanged
No Statin	1	0/1	0/1	D partial response
Alcohol Use	1	1/1	0/1	A unchanged clinically
No Alcohol	3	3/5	2/3	B, C improved; D unchanged
RCRI Class II	4	5/6	2/4	All patients classified as Class II; clinical improvement in B and C only
ARISCAT Score ≥ 26	3	4/5	1/3	Patients A, B, D; clinical improvement only in B
ARISCAT Score < 26	1	1/1	1/1	Patient C; clinically improved
Baseline mRS ≥ 3 (Moderate-Severe Disability)	2	3/4	1/2	C and D; C improved, D unchanged
Baseline mRS < 3 (Mild Symptoms/Independent)	2	3/3	1/2	A unchanged clinically; B improved
Follow-Up Interval < 10 weeks	2	3/4	1/2	A (eight weeks) radiographic success, no clinical change; D (six weeks) radiographic failure
Follow-Up Interval ≥ 10 weeks	2	2/2	2/2	B (10 weeks) and C (12 weeks) radiographic and clinical success

Follow-up ranged from six to 12 weeks, during which radiographic and clinical improvements were generally synchronous. Variability in follow-up timing may have influenced outcome progression but did not alter overall recovery trends.

Individual Case Narratives

Case A (63-year-old male): The patient sustained head trauma when a parked car rolled backward, causing occipital impact on the headrest. He experienced a brief loss of consciousness (<1 minute; GCS 15). Initial CT scans revealed progressive bilateral frontoparietal CSDHs, left homogeneous hyperdense and right mixed-density laminar types, measuring 21 mm and 20 mm, respectively, without midline shift.

He developed right hemiparesis and cognitive slowing, prompting bilateral MMA embolization via the internal maxillary artery (IMA) using a nonselective technique (EMMA Grade 3).

At eight weeks post-EMMA, both hematomas had markedly regressed (left 7 mm, right 3 mm), with complete radiographic resolution of mass effect. However, the patient exhibited persistent cognitive decline despite improvement in hemiparesis, yielding an unchanged overall functional outcome (mRS 2 → 2). No recurrence was noted (Tables 1-2; Figure 1).

Case B (70-year-old female): The patient presented with a four-day history of right hemiparesis, right-sided sensory loss, and severe bifrontal headache (8/10). CT revealed a right frontoparietal mixed-density CSDH measuring 20 mm, accompanied by a 5 mm midline shift. Open evacuation was declined due to multiple comorbidities, including acute coronary syndrome (ACS), hypertension (HTN), and type 2 diabetes mellitus (T2DM). Bilateral MMA embolization via the IMA using a nonselective technique (EMMA Grade 3) was subsequently performed.

Post-embolization, the patient experienced gradual clinical improvement. By postoperative day eight - the longest inpatient stay among the cohort - her previously severe bifrontal headache had improved to mild intensity with analgesic therapy, and right-sided weakness had resolved.

At twelve weeks post-EMMA, follow-up non-contrast CT demonstrated near-complete hematoma resolution, with a residual thickness of 3 mm (≈85% reduction) and restoration of midline symmetry. The patient achieved full neurological recovery (mRS 3 → 1) with no radiographic or clinical recurrence (Tables 1-2; Figure 2).

Case C (86-year-old female)

The patient fell backward onto concrete, initially remaining conscious (GCS 15). Over subsequent days, she developed somnolence, dysarthria, and total dependence.

CT revealed bilateral frontoparietal mixed-trabecular CSDHs (≈ 20 mm each) with a 9 mm rightward midline shift. Given her frailty and comorbidities (atrial fibrillation on apixaban, HTN, dyslipidemia, pulmonary tuberculosis (PTB)), she underwent bilateral MMA embolization (EMMA Grade 3) via the IMA using a nonselective approach.

At eight weeks post-EMMA, CT demonstrated complete hematoma resolution and restoration of midline alignment, paralleled by marked improvement in sensorium and cognition (mRS 5 → 2). No recurrence occurred (Tables 1-2; Figure 3).

Case D (71-year-old female): The patient sustained a blow to the left frontotemporal area one month prior to admission, followed by progressive left hemiparesis and persistent headache. CT revealed a right frontoparietal mixed-laminar CSDH (30 mm) with a 5 mm leftward midline shift.

Right MMA embolization via the IMA was performed with superselective posterior-division catheterization distal to the sphenoidal branch, as the anterior division was absent.

At six weeks post-EMMA, CT showed only partial hematoma reduction (23 mm residual; ≈23% decrease) and partially improved midline shift. The patient continued to experience headache and unchanged functional status (mRS 1 → 1). Further intervention was declined by the family (Tables 1-2; Figure 4).

Results synthesis and analysis

Across all cases, radiographic success (≥50% reduction in hematoma thickness) was achieved in five of six hematoma sides (83.3%) and in three of four patients (75%), while clinical improvement was noted in three patients (75%) (Tables 4-6). One patient (Case D) demonstrated only partial radiographic and clinical response, primarily associated with posterior-only embolization. No neurological or embolic complications occurred.

Descriptive subgroup analyses highlight several patterns. Hematoma distribution and laterality influenced outcomes: bilateral hematomas (Patients A and B) showed consistent radiographic regression, with clinical improvement in Patient B only, whereas unilateral hematomas (Patients C and D) demonstrated mixed outcomes, with one radiographic failure and incomplete clinical recovery in Case D (Tables 5-6). Pre-EMMA characteristics, including Nakaguchi/Nomura classification, maximum thickness, and midline shift, also correlated with outcomes, with larger hematomas (≥20 mm) showing greater variability in radiographic success when embolization was incomplete (Tables 5-6).

**Table 5:** Subgroup patterns of pre-EMMA CSDH parameters (four patients, six hematoma sides) CSDH, chronic subdural hematoma; EMMA, embolization of the middle meningeal artery; MLS, midline shift; Radiographic Success = ≥50% reduction in hematoma thickness on follow-up non-contrast computed tomography; Clinical Improvement = ≥1-grade reduction in modified Rankin Scale or resolution of presenting neurological symptoms.

Subgroup	n	Radiographic Success (≥50%)	Clinical Improvement	Notes/Observations
Hematoma Distribution - Bilateral	4	4/4	2/4	Patients A and B; all sides had radiographic success; clinical improvement in B only
Hematoma Distribution - Unilateral	2	1/2	2/2	Patients C and D; C left: radiographic success and clinical improvement; D right: radiographic failure, clinical unchanged
Laterality - Left	3	3/3	2/3	A left, B left, C left; A unchanged clinically, B, C improved
Laterality - Right	3	2/3	2/3	A right, B right, D right; D posterior-only, radiographic failure, clinical unchanged
Location - Frontal/Parietal	6	5/6	4/6	All hematomas frontal/parietal; partial reduction in D right
Pre-EMMA Nakaguchi/Nomura - Hyperdense Homogeneous	1	1/1	0/1	A left; clinical outcome unchanged
Pre-EMMA Nakaguchi/Nomura – Mixed Densities Laminar	3	1/3	1/3	A right, C left, D right; D posterior-only embolization led to radiographic failure
Pre-EMMA Nakaguchi/Nomura – Mixed densities trabecular	2	2/2	2/2	B left and right; full radiographic success; clinical improved
Pre-EMMA Max Thickness ≥20 mm	6	5/6	4/6	All hematomas ≥20 mm; D right partial radiographic response, clinical unchanged
Pre-EMMA Max Thickness <20 mm	0	–	–	No hematomas <20 mm in cohort
Pre-EMMA MLS = 0 mm	1	1/1	0/1	A bilateral: no net shift
Pre-EMMA MLS ≤5 mm (→ Right)	2	2/2	1/2	B left/right: 5 mm → R, radiographic success; clinical improvement on right only
Pre-EMMA MLS ≤5 mm (→ Left)	1	0/1	0/1	D right: 3 mm → L; partial radiographic response, clinical unchanged
Pre-EMMA MLS >5 mm (→ Right)	1	1/1	1/1	C left: 9 mm → R; full radiographic and clinical improvement

**Table 6:** Subgroup patterns of middle meningeal artery morphometry and procedural parameters (four patients, six hematoma sides) BP, branch point of the middle meningeal artery; CSDH, chronic subdural hematoma; EMMA, embolization of the middle meningeal artery; MMA, middle meningeal artery; Origin-BP Length, distance from the middle meningeal artery origin to the branch point (mm); Catheter Distal Point, distance of microcatheter tip from the branch point (mm); Radiographic Success, ≥50% reduction in hematoma thickness on follow-up non-contrast computed tomography; Clinical Improvement, ≥1-grade reduction in modified Rankin Scale or resolution of presenting neurological symptoms.

Subgroup	n	Radiographic Success (≥50%)	Clinical Improvement	Notes/Observations
MMA Trunk Thickness ≥1.5 mm	3	3/3	2/3	A right, B left, C left; larger trunks; clinical improvement in B,C only
MMA Trunk Thickness <1.5 mm	3	0/3	0/3	A left, B right, D right; small trunks; posterior-only embolization led to radiographic failure and unchanged clinical outcome
Anterior Branch Thickness ≥1 mm	4	4/4	2/4	A left/right, B left, C left; clinical improvement in B,C only
Anterior Branch Thickness <1 mm	1	0/1	1/1	B right; incomplete embolization or small branch; clinical improvement in B only
Posterior Branch Thickness ≥1 mm	4	3/4	1/4	A left/right, B left, D right; posterior branch embolized completely for some; clinical improvement in B only
Posterior Branch Thickness <1 mm	2	1/2	1/2	B right, C left; partial or small posterior branches; C left improved clinically, B right failed radiographically
Origin-BP Length ≥40 mm	3	3/3	2/3	A right, B left, C left; longer proximal segment; clinical improvement in B,C sides
Origin-BP Length <40 mm	2	0/2	0/2	A left, B right; shorter segment; D excluded (posterior-only)
Catheter Distal Point ≥7 mm	3	3/3	1/3	A left, B right, C left; longer microcatheter advancement; clinical improvement in C only
Catheter Distal Point <7 mm	2	2/2	1/2	A right, B left; shorter catheter placement; B left improved clinically, A right unchanged
Access - Transfemoral	5	5/5	3/5	All except D; full embolization; clinical improvement in B,C sides
Access - Transradial	1	0/1	0/1	D right; posterior-only; radiographic failure and no clinical improvement
Duration ≥60 min	3	2/3	1/3	A bilateral (165 min), B bilateral (67 min), D right (80 min); longer procedures; clinical improvement in B side only
Duration <60 min	1	1/1	1/1	C left (45 min); shorter procedure; clinical improved
Branch Dominance - Anterior	2	2/2	1/2	A left/right; complete anterior + posterior embolization; clinical improvement in right only, left unchanged
Branch Dominance - Codominant	3	3/3	2/3	B left/right, C left; full embolization; clinical improvement in B,C sides
Branch Dominance - Posterior-only	1	0/1	0/1	D right; posterior-only embolization; radiographic failure and no clinical improvement
EMMA Grade 3 - Complete Anterior + Posterior	5	4/5	3/5	Full embolization; A left/right unchanged clinically; B,C improved; D excluded (posterior-only)
EMMA Partial/Superselective	1	0/1	0/1	D right; incomplete embolization; radiographic failure and no clinical improvement

MMA morphometry and procedural parameters further elucidated trends. Larger trunk diameters (≥1.5 mm), anterior/posterior branch thicknesses ≥1 mm, longer origin-BP lengths (≥40 mm), and greater catheter distal advancement (≥7 mm) were generally associated with higher rates of radiographic success; however, clinical improvement did not always occur, particularly in posterior branch or distal catheter placements. Conversely, smaller vessels, shorter origin-BP lengths, limited catheter advancement, posterior-only branch dominance, and partial/superselective embolization were consistently linked to reduced efficacy (Table 6). Procedural access (transfemoral vs. transradial) and embolization duration showed similar trends: longer procedures and transfemoral access generally coincided with complete embolization, though clinical improvement was not guaranteed in every case.

Patient demographics, comorbidities, and medication use (antiplatelets, anticoagulants, statins, alcohol) showed no consistent effect on radiographic or clinical response (Table 4). Risk stratification scores (Revised Cardiac Risk Index (RCRI), Assess Respiratory Risk in Surgical Patients in Catalonia (ARISCAT)) similarly did not predict outcome, though clinical improvement was limited to a subset of patients (B and C) despite uniform risk classification.

Overall, these exploratory subgroup analyses suggest that procedural completeness, MMA morphology, and catheter positioning play a central role in determining EMMA efficacy, while baseline patient factors appear less influential. Given the exploratory design and small sample, these findings should be viewed as hypothesis-generating rather than confirmatory.

## Discussion

In this single-center case series, EMMA was demonstrated to be a safe and technically feasible primary treatment for CSDH among older Filipino adults, even within the constraints of a public tertiary hospital setting. Consistent with growing international evidence, the majority of our patients achieved radiographic and clinical improvement, supporting EMMA as an effective minimally invasive alternative to conventional surgical evacuation in selected elderly individuals. The findings from the expanded subgroup analyses (Tables 4-6) further elucidate the clinical, radiologic, and procedural factors influencing EMMA outcomes.

Determinants of radiographic and clinical response

Radiographic failure was observed only in Patient D, who exhibited an atypical MMA anatomy characterized by the absence of an anterior branch, a small-caliber trunk (1.27 mm), and posterior-only embolization (Table 6). This combination likely limited embolic penetration into the vascular network sustaining the hematoma membranes. Salem et al. [11] identified MMA diameter < 1.5 mm as an independent predictor of EMMA failure, both clinically and radiographically, underscoring the importance of vessel caliber in achieving complete neovascular occlusion. Moreover, posterior-branch-only catheterization distal to the sphenoidal branch, as in this patient, has been linked to higher rates of incomplete embolization due to sparing of anterior dural feeders. These anatomic constraints, coupled with a relatively short imaging follow-up of six weeks, plausibly explain the observed radiographic persistence.

Our findings reinforce that adequate embolic coverage of both anterior and posterior MMA branches is critical to treatment success (Tables 5-6). Conversely, superselective posterior-only approaches-though safer in certain contexts-may risk incomplete devascularization of the hematoma capsule. Pre-procedural angiographic mapping of MMA dominance, branching pattern, and origin-branch-point (BP) distance is therefore indispensable to optimize catheter positioning and maximize embolic efficacy. In this cohort, larger MMA trunks (≥ 1.5 mm), codominant or anterior-dominant branch patterns, longer origin-BP lengths (≥ 40 mm), and more distal catheter placement (≥ 7 mm from BP) were associated with complete embolization and favorable clinical recovery (Table 6).

Radiographic success has also been correlated with baseline hematoma thickness ≥20 mm (i.e., smaller pre-treatment hematomas were associated with lower recurrence-free success when EMMA was performed as the primary intervention) and with longer imaging follow-up intervals, as resolution rates improved from 64.7% at ≤90 days to 89.7% beyond 90 days [11]. In our cohort, all hematomas measured ≥20 mm pre-EMMA, and radiographic follow-up ranged from six to twelve weeks. All patients followed for ≥10 weeks achieved at least partial resolution, while those evaluated earlier often demonstrated only radiographic improvement without parallel clinical recovery. These findings support the notion that EMMA promotes progressive rather than immediate hematoma resorption, emphasizing the need for sufficient post-procedure surveillance before judging treatment efficacy.

Ongoing bleeding and mechanistic insights from radiographic failure

In Patient D, post-EMMA NCCT paradoxically showed increased hyperdensities within the subdural collection, raising the possibility of ongoing low-grade bleeding or exudative activity despite technically successful embolization. This paradox can be explained by incomplete devascularization secondary to unfavorable vascular anatomy. The small-caliber MMA trunk and absent anterior branch limited embolic distribution to the posterior dural territory, leaving the anterior neovascular supply largely intact. Residual perfusion of the hematoma membranes through unembolized anterior or collateral feeders could have sustained slow rebleeding or proteinaceous exudation, leading to increased post-EMMA density.

Furthermore, trauma-induced angiogenesis and collateral recruitment may have contributed to persistent perfusion. De novo meningo-pial or diploic anastomoses can develop after trauma, allowing indirect revascularization of the hematoma capsule even after apparent angiographic occlusion. Watchmaker et al. [12] described a post-traumatic meningo-pial connection between the MMA and middle cerebral artery via the anterior temporal branch, an analogous mechanism that may underlie the partial radiographic failure observed in this case.

Another plausible mechanism is delayed collateral reperfusion through unrecognized meningeal-pial or orbital channels, a process described in recurrent or refractory CSDH after posterior-only or proximal embolization. Senol et al. [13] reported a case of hematoma recurrence secondary to collateral revascularization through contralateral meningeal and orbital feeders following unilateral EMMA. In the present case, contralateral pre-EMMA angiography was not performed, as the lesion was confined to the right hemisphere; thus, potential cross-midline or orbital collateral recruitment could not be fully assessed. The combination of a hypoplastic MMA, limited embolic penetration, and possible collateral reperfusion therefore provides a coherent pathophysiologic explanation for the apparent ongoing hemorrhagic activity in Patient D.

An additional consideration is the presence of an anatomical variant of the MMA itself. In approximately 0.5-1.5% of angiographic or imaging series, the MMA, or more commonly its anterior branch, originates from the ophthalmic artery rather than the maxillary artery [14]. This variant is often associated with absence or hypoplasia of the foramen spinosum, which may limit endovascular access and embolization completeness [14]. In such cases, embolization via the external carotid system may fail to reach the anterior dural territory, particularly if the ophthalmic-origin anterior branch remains unrecognized [14]. As a result, persistent anterior membrane perfusion may continue despite an otherwise technically successful procedure, reinforcing the importance of pre-procedural imaging to assess for vascular variants in patients with radiographic or clinical signs of embolization failure.

From a procedural standpoint, strategies such as proximal trunk embolization or the use of low-viscosity liquid embolic agents may enhance distal penetration and achieve more complete neovascular occlusion, provided angiographic safety with respect to ophthalmic and petrosal anastomoses is ensured. This case underscores the importance of detailed angiographic mapping, individualized embolization planning, and close radiologic follow-up to detect and address early signs of incomplete occlusion.

Influence of medications, comorbidities, and risk stratification

Anticoagulant and antiplatelet therapy, present in three of four patients, did not appear to adversely influence outcomes (Table 4). Similar to prior studies [11,15], no reduction in embolization success or clinical recovery was observed, likely because the embolic material mechanically isolates the neomembrane vasculature from systemic circulation, minimizing the impact of systemic coagulation status on local hemostasis.

Comorbidities, particularly cardiovascular disease, were common (75% of the cohort) yet did not demonstrate a consistent relationship with outcome. Patients with HTN, ischemic heart disease, or prior cerebrovascular events achieved comparable radiographic regression to those without such conditions. This suggests that EMMA efficacy may be preserved even in patients with moderate vascular or systemic disease, reinforcing the concept that the success of embolization depends more on the technical completeness of neovascular occlusion than on global hemodynamic or metabolic status. However, comorbidities may exert subtle, indirect effects on recovery trajectories, through mechanisms such as impaired cerebral autoregulation, microvascular fragility, or delayed hematoma resorption, potentially contributing to the discrepancy occasionally observed between radiographic and clinical outcomes.

Risk stratification indices paralleled these findings. All patients were classified as RCRI Class II, and three as ARISCAT ≥26, reflecting moderate predicted perioperative risk. Despite this uniform risk profile, only two patients (B and C) demonstrated clinical improvement, highlighting that traditional perioperative risk tools may not capture the local microvascular and inflammatory factors governing EMMA efficacy. Rather than predicting procedural success, these indices may better reflect systemic resilience or recovery potential after embolization. Their limited discriminative value in this context underscores the need for neurointervention-specific risk models that integrate angiographic and hemodynamic parameters alongside conventional systemic risk factors.

Interestingly, Patient A, on statin therapy and reporting mild alcohol use, achieved bilateral radiographic success despite theoretical risk factors for platelet dysfunction or altered vascular reactivity. While chronic alcoholism has been associated with platelet dysfunction and reduced embolization efficacy [16], this patient’s normal coagulation profile and technically complete bilateral embolization likely mitigated such risks. This further supports the notion that local embolic isolation can offset systemic vulnerabilities when procedural completeness is achieved.

In summary, the interplay between comorbidities, pharmacologic therapy, and procedural factors appears multifactorial and context-dependent. Systemic disease burden and standard surgical risk indices did not predict EMMA outcomes in this small series, emphasizing that technical factors-including embolization completeness, vascular morphology, and branch coverage-remain the dominant determinants of success. Future studies incorporating physiologic and microcirculatory assessments may clarify how specific comorbidity profiles influence recovery kinetics and recurrence risk following EMMA.

Trauma, vascular remodeling, and functional outcome

The impact of trauma on EMMA outcomes remains underexplored. In our cohort, both post-traumatic patients (A and D) demonstrated slower or incomplete radiographic resolution and minimal change in mRS scores compared with the non-traumatic group (Table 4). This may reflect trauma-induced vascular remodeling and persistent meningeal-brain anastomoses, which can maintain collateral perfusion to the hematoma membrane even after MMA embolization.

As previously discussed, Watchmaker et al. (2024) [12] described a post-traumatic meningo-pial connection between the MMA and the middle cerebral artery via the anterior temporal branch, an anatomic variant that can persist or reconstitute following EMMA. This same mechanism, earlier implicated in the radiographic failure of Patient D, likely also contributed to the slower hematoma regression observed in other trauma-related cases. Additionally, persistent mass effect, preexisting cerebrovascular disease, and post-concussive symptoms may have compounded the limited functional recovery despite adequate radiographic improvement.

Contextualizing results within major randomized trials

The current findings are consistent with results from three landmark multicenter randomized trials-EMBOLISE, MAGIC-MT, and STEM-that have recently established the clinical value of EMMA in CSDH management. The EMBOLISE trial demonstrated that adjunctive EMMA significantly reduced hematoma recurrence or progression requiring repeat surgery (4.1% vs. 11.3%) compared with standard surgical care, without an increase in major neurological events [7]. Similarly, the MAGIC-MT trial, which evaluated EMMA in both surgical and conservative contexts, reported lower rates of symptomatic recurrence or progression (7.2% vs. 12.2%) in the embolization arm [8]. The STEM trial, employing the non-adhesive embolic agent Squid, further reinforced these outcomes by showing a reduced overall treatment failure rate (15.2% vs. 39.2%) when EMMA was added to standard management [6].

Although our cohort was small and drawn from a resource-limited tertiary government hospital, our findings parallel these international results. The comparable rates of radiographic resolution and functional recovery observed here indicate that EMMA’s therapeutic advantage is reproducible across different healthcare settings, even when performed as a primary therapy rather than adjunctive to surgery.

Procedural and institutional considerations

All procedures were performed under conscious sedation using PVA particles as the embolic agent, following a standardized protocol adapted from the Barrow algorithm [2]. Procedural safety was excellent, with no intraoperative complications or periprocedural neurological decline. Minor puncture-site hematomas occurred in two cases and resolved spontaneously without intervention.

Access route and procedural duration demonstrated subtle associations with outcome. Transfemoral access, used in five of six treated sides, provided stable catheter support and more complete anterior-posterior branch embolization, correlating with higher rates of radiographic success. In contrast, the single transradial case was technically limited to posterior-only embolization and resulted in both radiographic and clinical non-response. Longer procedures (≥60 minutes) typically reflected greater anatomic complexity or bilateral treatment rather than inefficiency, and while these cases achieved complete embolization, clinical improvement did not always parallel technical success-suggesting that duration reflects procedural complexity rather than efficacy per se.

Hematoma distribution also influenced procedural planning and outcomes. Bilateral cases (Patients A and B) required sequential dual-sided embolization under a single session, achieving consistent radiographic regression, whereas unilateral cases (Patients C and D) showed greater variability, particularly when embolization was incomplete or posterior-dominant. These trends highlight the importance of individualized catheter positioning and branch selection based on laterality and vascular dominance.

From an institutional standpoint, all procedures were conducted in a government tertiary hospital setting with limited dedicated neurointerventional infrastructure, yet achieved uniform safety and procedural reproducibility. Post-EMMA hospitalization was short in most cases (two to four days), reflecting rapid recovery and minimal procedural morbidity. The longest post-EMMA stay (8 days) occurred in a single patient who had a persistent severe headache, managed conservatively with analgesics and without neurological sequelae or radiographic deterioration.

Collectively, these findings underscore that EMMA can be safely and effectively implemented in tertiary centers, even within resource-constrained environments, when supported by appropriate operator expertise, case selection, and protocol standardization. Procedural completeness, rather than duration, access route, or hematoma distribution, remains the key determinant of success, while optimization of access strategy and post-embolization care may further improve patient comfort and institutional efficiency.

Implications for low- and middle-income settings

In resource-limited healthcare systems such as the Philippines, where neurosurgical capacity and postoperative support may be constrained, EMMA offers a lower-risk, cost-conscious, and anesthetically sparing treatment pathway for elderly patients with multiple comorbidities. Importantly, this modality aligns with the increasing global shift toward endovascular and outpatient-based neurosurgical care.

Broader adoption of EMMA in similar low- and middle-income contexts could help reduce CSDH-related morbidity and mortality, particularly among high-risk geriatric populations. The convergence between our real-world results and those of the EMBOLISE, MAGIC-MT, and STEM trials underscores that even within constrained healthcare systems, EMMA can achieve outcomes consistent with global standards-supporting its role as a scalable and sustainable strategy in modern CSDH management.

Limitations

This case series has several limitations. First, the small sample size (n = 4) limits statistical power and generalizability, and findings should be interpreted as exploratory. Second, the short follow-up period (6-12 weeks) may not fully capture late recurrences or long-term clinical outcomes. Third, the absence of a control or surgical comparison group precludes definitive conclusions about relative efficacy versus conventional burr-hole drainage. Fourth, radiographic and clinical assessments were performed at variable follow-up intervals, which may have introduced timing-related bias. Finally, the study reflects the experience of a single tertiary government center with specific procedural resources, and outcomes may differ in other institutions with varying neurointerventional capacity. Future multicenter, prospective studies with standardized imaging and longer follow-up are needed to validate these findings and guide practice in low- and middle-income country settings.

## Conclusions

EMMA can serve as a safe and practical primary treatment for CSDH in older adults, offering a lower-risk alternative to burr-hole drainage, especially for patients unfit for general anesthesia. Outcomes in this small Philippine case series suggest that procedural completeness and vessel anatomy play key roles in success.

## References

[1] Winn HR (2022). Youmans and Winn Neurological Surgery. 8th Edition.

[2] Rudy RF, Catapano JS, Jadhav AP (2023). Middle meningeal artery embolization to treat chronic subdural hematoma. Stroke Vasc Interv Neurol.

[3] Shankar J, Alcock S, Milot G (2025). Embolization of middle meningeal artery for chronic subdural hematoma: do we have sufficient evidence?. Interv Neuroradiol.

[4] Shankar J, Kaderali Z (2023). Grading scale for embolization of middle meningeal artery for chronic subdural hematoma. Can J Neurol Sci.

[5] Rojas-Villabona A, Mohamed S, Kennion O, Padmanabhan R, Siddiqui A, Prasad M, Mukerji N (2023). A systematic review of middle meningeal artery embolization for minimally symptomatic chronic subdural haematomas that do not require immediate evacuation. Brain Spine.

[6] Fiorella D, Monteith SJ, Hanel R (2025). Embolization of the middle meningeal artery for chronic subdural hematoma. N Engl J Med.

[7] Davies JM, Knopman J, Mokin M (2024). Adjunctive middle meningeal artery embolization for subdural hematoma. N Engl J Med.

[8] Liu J, Ni W, Zuo Q (2024). Middle meningeal artery embolization for nonacute subdural hematoma. N Engl J Med.

[9] Khu KJ, Chan KI, Pascual JS, Hernandez MA (2024). Neurosurgery in the elderly: findings from a cohort in the Philippines. J Clin Neurosci.

[10] Mathew G, Sohrabi C, Franchi T, Nicola M, Kerwan A, Agha R (2023). Preferred Reporting Of Case Series in Surgery (PROCESS) 2023 guidelines. Int J Surg.

[11] Salem MM, Kuybu O, Nguyen Hoang A (2023). Middle meningeal artery embolization for chronic subdural hematoma: predictors of clinical and radiographic failure from 636 embolizations. Radiology.

[12] Watchmaker JM, Sisti JA, Shigematsu T (2024). Occult middle meningeal artery to middle cerebral artery anastomosis associated with prior trauma. BMJ Case Rep.

[13] Senol YC, Asghariahmadabad M, Cooke DL, Savastano LE (2024). A case of recurrent subdural hematoma after unilateral MMA embolization that resolved after contralateral MMA embolization. Interv Neuroradiol.

[14] Akdemir Aktaş H, Mine Ergun K, Tatar İ, Arat A, Mutlu Hayran K (2022). Investigation into the ophthalmic artery and its branches by superselective angiography. Interv Neuroradiol.

[15] Housley SB, Monteiro A, Donnelly BM (2022). Statin versus nonstatin use in patients with chronic subdural hematomas treated with middle meningeal artery embolization alone - a single-center experience. World Neurosurg.

[16] Orscelik A, Senol YC, Bilgin C (2023). Middle meningeal artery embolization without surgical evacuation for chronic subdural hematoma: a single-center experience of 209 cases. Front Neurol.

